# Novel *WNT10A* variant in a Japanese case of nonsyndromic oligodontia

**DOI:** 10.1038/s41439-023-00230-3

**Published:** 2023-01-26

**Authors:** Junya Adachi, Yoshihiko Aoki, Hiroto Izumi, Takeshi Nishiyama, Atsuo Nakayama, Masatoshi Sana, Kyoko Morimoto, Atsuo Kaetsu, Takamasa Shirozu, Eriko Osumi, Michiko Matsuoka, Eri Hayakawa, Nasel Maeda, Junichiro Machida, Toru Nagao, Yoshihito Tokita

**Affiliations:** 1 Department of Oral and Maxillofacial Surgery, Toyohashi Municipal Hospital, Toyohashi, Japan; 2 Department of Maxillofacial Surgery, School of Dentistry, Aichi-Gakuin University, Nagoya, Japan; 3 Department of Disease model, Institute for Developmental Research, Aichi Developmental Disability Center, Kasugai, Japan; 4 University of Occupational and Environmental Health, Fukuoka, Japan; 5 Nagoya City University, Nagoya, Japan; 6 Department of Cellular Pathology, Institute for Developmental Research, Aichi Developmental Disability Center, Kasugai, Japan; 7 Nagoya Orthodontic Clinic, Nagoya, Japan; 8 Department of Otorhinolaryngology, Toyota Memorial Hospital, Toyota, Japan; 9 Department of Oral and Maxillofacial Surgery, Toyota Memorial Hospital, Toyota, Japan

**Keywords:** Diseases, Developmental biology, Genetic variation

## Abstract

Congenital tooth agenesis is one of the most common anomalies in humans. Many genetic factors are involved in tooth development, including *MSX1*, *PAX9*, *WNT10A*, and *LRP6*. Thus, mutations in these genes can cause congenital tooth agenesis in humans. In this study, we identified a novel nonsense *WNT10A* variant, NM_025216.3(WNT10A_v001):c.1090A > T, which produces a C-terminal truncated gene product, p.(Lys364*), in a sporadic form of congenital tooth agenesis. The variant was not found in the healthy parents and thus was considered to cause congenital tooth agenesis in the case.

Congenital tooth agenesis (TA) is classified by the number of missing teeth: hypodontia is defined as five or fewer missing teeth, excluding the wisdom teeth, while oligodontia is defined as six or more missing teeth^1^. Congenital TA is the most frequent dental anomaly in humans, with a frequency of 6.8% (95% confidence interval: 6.1–7.7%) for hypodontia and 0.1% (95% confidence interval: 0.04–0.3%) for oligodontia in the Japanese population. We previously demonstrated that the sibling recurrence risk ratio of oligodontia is 43.8%, suggesting that oligodontia exhibits a dominant mode of inheritance in most cases^2–6^.

Robust tooth development requires a variety of growth factors produced by the oral ectodermal epithelium, such as fibroblast growth factors, bone morphogenic proteins, and the gene *WNT10A*. *WNT10A* is the most frequent cause of human nonsyndromic TA (STHAG4; MIM 150400), including in the Japanese population^6^. The WNT signal stabilizes intracellular β-catenin, which activates lymphoid enhancer factor/T-cell factor protein. In tooth germ cells, WNT/β-catenin signaling induces the expression of essential transcription factors for human tooth development, such as *MSX1, PAX9, RUNX2*, and *BMP4*^7–10^. However, the genetic causes remain unknown in more than 50% of human TA cases^11–17^. Therefore, we performed whole-exome sequencing analysis to explore the genetic cause of an individual case.

The patient was a 21-year-old woman with a tooth missing at 17, 13, 12, 22, 23, 27, 28, 38, 48 (FDI tooth numbering system), confirmed by X-ray photography (Fig. 1A). The patient had no systemic abnormality except for TA, including in the crown morphology of the other teeth or the jawbone. No other family members had any abnormalities in tooth number (Fig. 1B).

**Fig. 1:**
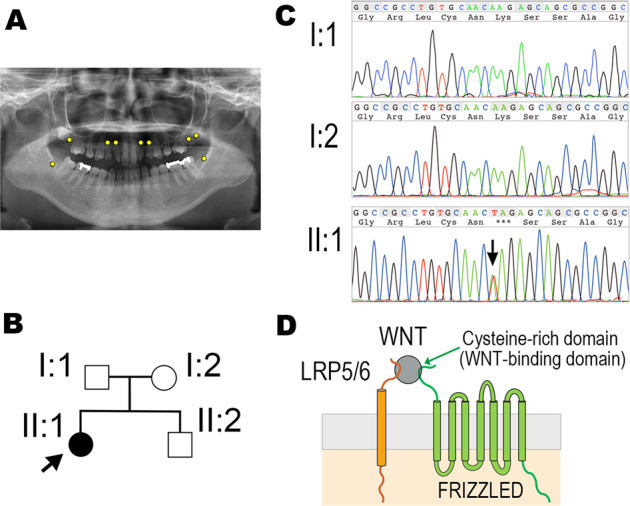
Oral phenotype, pedigree trees, and nucleotide substitution in *WNT10A*. **A** Orthopantomograms with the patient’s missing teeth indicated by yellow circles. **B** Pedigree tree of the patient’s family. The proband is indicated by an arrow. Affected individuals are depicted by black symbols, and unaffected family members are shown by white symbols. **C** Sanger sequencing showing the c.1090 A > T nucleotide substitution in exon 4 of *WNT10A*. **A** Electropherogram of I:1 (father), I:2 (mother) and II:1 (patient). **D** Schematic diagram of WNT, LRP5/6, and FRIZZLED molecular complexes. WNT ligands bind to two transmembrane proteins: LRP5/6 and FRIZZLED. The cysteine-rich domain (CR-domain) of FRIZZLED is a binding domain for WNT ligands.

According to the manufacturer’s protocol, genomic DNA was extracted from 2 ml of saliva with the Oragene® DISCOVER kit. Then, whole-exome sequencing (WES) was performed with the proband’s genomic DNA. A novel heterozygous *WNT10A* variant, NM_025216.3(WNT10A_v001):c.1090 A > T, was identified in the proband (II:1). With a specific primer set (5′-CGCCGACCTGGTCTACTTC-3′, 5′-CTTCGCAGACCACGAAACAG-3′), the nucleotide substitution was confirmed by polymerase chain reaction and Sanger sequencing, revealing that the variant was not shared with the healthy parents (Fig. 1C). The variant was not in the gnomAD online database (https://gnomad.broadinstitute.org/).

WNT ligands, including WNT10A, are cysteine-rich morphogens that can interact with the Frizzled (FZD) receptor and LDL receptor-related protein 5/6 (LRP5/6) (Fig. 1D). With crystal structure analyses, human WNT3 has been demonstrated to form a complex with the Cys-rich domain of mouse FZD8 (6AHY)^18^. Thus, the PyMOL Molecular Graphics System (PyMOL) was used to graphically predict the effect of the truncated form of the WNT10A variants detected by WES in three dimensions. Specifically, the anti-parallel beta-sheet secured by disulfide bonds in the C-terminus of WNT ligands is a crucial region for binding to the FZD receptor. The nucleotide substitution identified in the current case resulted in a premature stop codon at nucleotide 1090. Thus, the product of the WNT10A gene lacks the C-terminus, resulting in the variant p.Lys364*. Because the amino acid sequence, especially the arrangement of cysteine residues in the C-terminus, is highly conserved between WNT10A and WNT3 (Fig. 2Aa), they share the same higher-order structure and, consequently, the same biological function. Thus, it is plausible that the biological function of the C-terminal domain of WNT10A is to interact with FZD receptors (Fig. 2Ab–e).

**Fig. 2:**
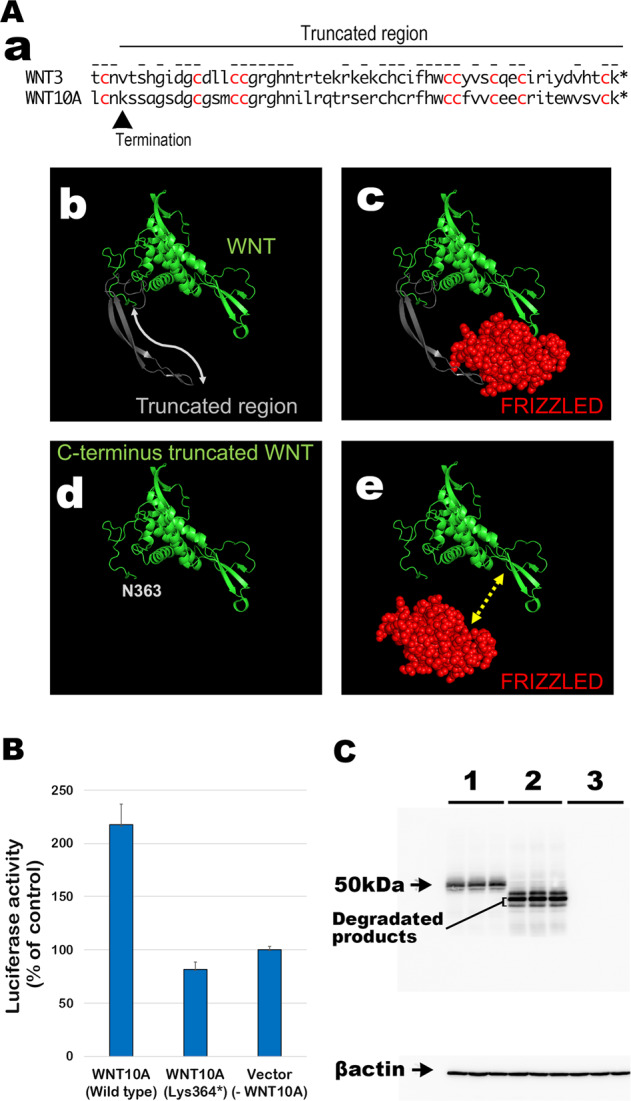
Structural models and biological activity of wild-type WNT10A and the p.Lys364* variant. **A, a** Amino acid sequence of the C-termini of human WNT3 and WNT10A. The truncated region is indicated by the line. The conserved amino acid residues are marked with a dash above the sequence and the cysteine residues are in red. **b** Structural model of WNT10A. The truncated region in the C-terminus anti-parallel beta-sheet of the WNT variant is indicated in silver gray and by the double-headed arrow. **c** The structure of WNT is indicated by green ribbon model and the CR-domain of FRIZZLED is shown as a red sphere model. The models were created with PyMOL Molecular Graphics System. The CR-domain of FRIZZLED interacts with the WNT ligand via the C-terminus beta-sheet structure. **d**, **e** The C-terminal deletion variant of WNT10A lacks an interaction arm and does not bind the CR-domain of FRIZZLED. **B** Luciferase assay for WNT/beta-catenin signaling. The WNT10A p.Lys364* variant has lost biological activity relative to the negative control level. **C** Western blotting of WNT10A and the variant from the transfectant cell lysate (upper panel); beta-actin served as a control (lower panel). The experiment was performed in triplicate. Lane 1, wild-type human WNT10A; Lane 2, human WNT10A p.Lys364* variant; Lane 3, mock transfection with pcDNA3.1. The degradation product is detected in the p.Lys364* WNT10A variant protein samples. The beta-actin control experiment confirmed the equality of expression levels in each sample.

We performed a luciferase assay in 12-well plates with a stable PC3 reporter cell line of TCF/LEF-responsive luciferase. The following expression vectors were cotransfected: LRP6, FRIZZLED4, and WNT10A (500 ng/well). The cells with C-terminal–truncated WNT10A (p. Lys364*) exhibited significantly decreased WNT/β-catenin signaling compared with the level of the wild-type control (Fig. 2B). Western blot analysis for the wild-type and p. Lys364* variant was then performed to assess the variant protein expression levels and molecular instability. Degraded immunopositive bands were weakly detected in the WNT10A variant lanes, indicating that truncated WNT10A would be slightly more unstable than the wild-type in PC3 cells (Fig. 2C). In conclusion, we identified a novel nucleotide substitution in the WNT10A gene as the genetic cause of congenital TA.

## HGV Database

The relevant data from this Data Report are hosted at the Human Genome Variation Database at 10.6084/m9.figshare.hgv.3273.
